# Bibliometric analysis of VR interventions in cancer research based on multiple databases: mapping research hotspots and developmental trends

**DOI:** 10.3389/fonc.2026.1794020

**Published:** 2026-10-01

**Authors:** Ruihan Wang, Yujie Zhang, Lidong Fang, Shuoqi Li

**Affiliations:** School of Sports Science, Nantong University, Nantong, Jiangsu, China

**Keywords:** cancer, CiteSpace, digital health, pain management, virtual reality

## Abstract

**Background:**

Virtual reality (VR) has become an increasingly popular tool in cancer care over the years. It shows promise in areas like pain management, psychological support, surgical training, and rehabilitation. Even though more studies are being done, there has not been a thorough analysis of the research trends in this field. This study looks at how VR interventions in cancer research have evolved over the past two decades, identifies research hotspots, and explores future directions.

**Methods:**

We collected publications related to VR in cancer research from the Web of Science Core Collection and Pubmed, covering the period from 2005 to 2025. Using VOSviewer and CiteSpace software, we performed bibliometric and visual analyses. These included examining publication trends, contributions by countries and institutions, author collaboration networks, journal impact, keyword co-occurrence, and citation bursts.

**Results:**

A total of 1,259 publications from 110 countries were included. The number of publications fluctuated over time but generally increased, reaching its highest point in 2024 with 184 articles. The United States and China were the most active countries. Jordan had the highest average number of citations per paper. Among institutions, McGill University published the most papers, followed by the University of Toronto. The most productive journal was Surgical Endoscopy and Other Interventional Techniques, whilst Cancers and Supportive Care in Cancer showed the fastest growth. Main research themes included pain management, anxiety reduction, breast cancer, and surgical training. Emerging trends involve the combination of augmented reality (AR), digital health, and artificial intelligence (AI) with VR.

**Conclusion:**

This study shows that VR research in cancer care is expanding worldwide, with most contributions coming from North America and Europe. Pain management remains the most common application. New technologies such as AR, AI, and telemedicine are becoming more integrated into VR interventions.

## 1 Introduction

Cancer is a critically harmful illness and significantly threatens the health of millions of individuals (1). Abnormal cell proliferations that give rise to new organisms are known as cancer. The new organisms commonly manifest as atypical local tissue masses in the body. Meanwhile, biomedical reports define cancer as an illness due to carcinogenic factors-influenced severe disorders in cell growth regulation (2). Cancer patients present with various symptoms, including anxiety (3), depression (3), pain (4), fatigue (5), and cognitive impairment (6). Insufficient psychological management of the symptoms may decrease quality of life (7). Cancer prognosis, where elevated cancer-specific mortality and diminished survival rate may also arise from inadequate symptom management (8). Given the inability of routine cancer treatment to fully address patient psychological distress and persistent symptom burden, complementary intervention strategies are required to optimise comprehensive cancer care.

Individuals utilising virtual reality (VR) equipment can interact with a computer-generated three-dimensional (3D) virtual environment in real-time. Achieving full immersion through VR commonly involves employing head-mounted devices (9). Nevertheless, other devices can also be utilised. For example, non-immersive equipment allows limited immersion, whilst semi-immersive tools permit semi-partial immersion within a defined physical space (9). The VR technology has demonstrated potential to support cancer patients (10), where reports have demonstrated its applicability at different phases of the treatment protocol. Furthermore, numerous potential utilisation and effects on psychological, physiological, and biological variables have also been observed (11). During chemotherapy infusion and hospitalisation, VR also offers emotional management benefits (11). Another research indicated the analgesic effects of VR during painful procedures (12), particularly by engaging patients in virtual surroundings that are distracting and relaxing. In another study, VR-based interventions were implemented as personalised comprehensive rehabilitation games to improve specific physical and cognitive functions post-cancer treatment (13). The VR is gaining attention as a solution for conveying medical data, enhancing comprehension of complex treatments, such as radiotherapy and surgery, and decreasing anxiety to certain treatments (14). Moreover, VR presents substantial potential in the healthcare system. The technology can reduce drug demand (15), shorten hospitalisation period (16), and improve treatment compliance (17). Therefore, utilising VR in healthcare can lead to noticeable cost savings and resource allocation improvements. Existing studies have confirmed the multiple clinical advantages of VR and support its wide application in cancer supportive care.

Although a growing body of reviews has examined VR applications in cancer care, their scope and methods differ substantially from the present study. An umbrella review synthesised evidence from 26 systematic reviews and meta-analyses, confirming significant effects of VR on anxiety, depression, and pain, but did not map the structural evolution or collaborative networks of the field (18). A scoping review identified 23 primary studies and categorised VR purposes into distraction, training, and rehabilitation, yet it provided no quantitative analysis of publication trends or geographic distribution (19). Systematic reviews with meta-analyses have quantified VR effects on symptom management but are constrained by narrow inclusion criteria focused on specific outcomes or cancer types (20, 21). These prior studies share three key limitations. First, they rely on qualitative synthesis or pooled effect sizes rather than quantitative mapping of the entire literature landscape. Second, they do not identify collaborative networks amongst countries, institutions, and authors. Third, they lack temporal analysis of emerging research hotspots and frontier trends. Consequently, the field still lacks a comprehensive, data-driven overview of how VR cancer research has developed globally, which topics have gained or lost momentum, and where future research priorities should lie. In this study, the latest advancements of VR interventions in cancer research were assessed with bibliometric analysis. In literature studies, bibliometric analysis presents a quantitative scientific approach. Literature review is vital amongst scientific researchers. Nonetheless, failing to apply appropriate reading and assessment approaches would result in the inability to navigate the substantial amount of data available. Applying literature visualisation on VR intervention in cancer studies enables the rapid comprehension of the literature development trend in the field. High-quality authors and high-yield research institutions can be targeted with the method. Furthermore, the rapid determination of literature research topics with significant credibility and quality is achievable with literature visualisation, potentially accelerating the introduction of scientific research (22). VOSviewer and Citespace are examples of the software applicable for displaying visual maps (23).On this basis, this study adopts bibliometric methods to systematically analyse VR-related cancer literature published from 2005 to 2025. This study aims to clarify the research status, hot topics and development trends of this field, and further summarise the mechanism of VR intervention on cancer pain.

To address these gaps, this study employs bibliometric analysis and visualisation techniques to map VR cancer research from 2005 to 2025. This study has four specific objectives. It first examines the annual publication trends and geographical distribution of VR cancer research. It then identifies the most productive countries, institutions, authors, and journals, along with their collaboration patterns. It further analyses the core research hotspots and their temporal evolution. Finally, it detects emerging frontiers for future investigation. By achieving these objectives, this study provides a macroscopic and quantitative overview that complements existing qualitative reviews and meta-analyses. The visualised results can help researchers identify potential collaborators, recognise understudied areas, and plan subsequent investigations. In addition, this study summarises the mechanisms underlying VR-induced analgesia, offering evidence to support the clinical application and promotion of VR as a cancer adjuvant therapy.

This study outlined the country, institution, author, source journal, keyword co-occurrence, and co-citation distributions of publications extracted from the Web of Science and Pubmed. Only articles published from 2005 to 2025 were involved. This study also provided an extensive collation and summarised keywords based on keyword bursts, which enabled the identification of research hotspots or future trends. Furthermore, a summary of the principles and mechanisms of how VR interventions affect cancer pain is included. The information provided in this study can significantly facilitate researchers and aid experts and novices in recognising the range of their disciplines, pinpointing new topics of interest, and visually planning subsequent investigations.

## 2 Materials and methods

### 2.1 Data source and search strategy

This study is a bibliometric analysis rather than a systematic review, so it does not require PROSPERO registration, dual independent screening, or risk of bias assessment as specified by PRISMA guidelines. The literature screening and data extraction were conducted by two researchers and verified by a third researcher to ensure accuracy. The WOS is amongst the most reliable and complete databases globally. The substantial content and high-quality academic journals offered by the database make it amongst the most frequently employed databases for bibliometric assessments. Therefore, this study employed the Web of Science Core Collection (WoSCC) and Pubmed as the database. The WoSCC provides comprehensive citation metadata and consistent bibliographic records, which are essential for co-citation and bibliographic coupling analyses. PubMed was included to cover biomedical journals that may be underrepresented in WoSCC, ensuring adequate coverage of the oncology literature. The combination of WoSCC and PubMed has been widely adopted in recent bibliometric studies of digital health interventions and was deemed sufficient to capture the core literature on VR in cancer care.

Two investigators in this study simultaneously performed a literature search with the Science Citation Index Expanded (SCIEXPANDED) as the citation database. Comprehensiveness and accuracy of the retrieved data were ensured with the utilisation of SCIEXPANDED. During the literature search stage, the search keywords “TS = ((“Virtual reality” or VR) and (Cancer or Malignancy or Tumour or Neoplasm)) and document type = (ARTICLE OR REVIEW) and language = (English)” were applied. Only publications between 1^st^ January 2005 and 31^st^ August 2025 were considered. All valid bibliographic information, including publication year, title, author name, nationality, affiliation, abstract, keywords, and journal name, were then stored in plain text format in the WoSCC and Pubmed database.

The inclusion criteria were as follows: (1) publications focused on virtual reality interventions in cancer research; (2) article types limited to original articles and reviews; (3) publications written in English; (4) publication date between January 1, 2005 and August 31, 2025. The exclusion criteria included: (1) conference abstracts, editorials, letters, and book chapters; (2) publications unrelated to VR or cancer; (3) duplicate records across the two databases. The initial search retrieved 1,847 records from WoSCC and 612 records from PubMed, yielding 2,459 records in total. After removing 523 duplicates, 1,936 records remained. Following title and abstract screening, 567 records were excluded for not meeting the inclusion criteria, and 110 full-text articles were further excluded after full-text assessment. Finally, 1,259 publications were included in the bibliometric analysis. A study selection flow diagram was added as **Figure 1** to illustrate the study selection process. (**Figure 1a**).

**Figure 1:**
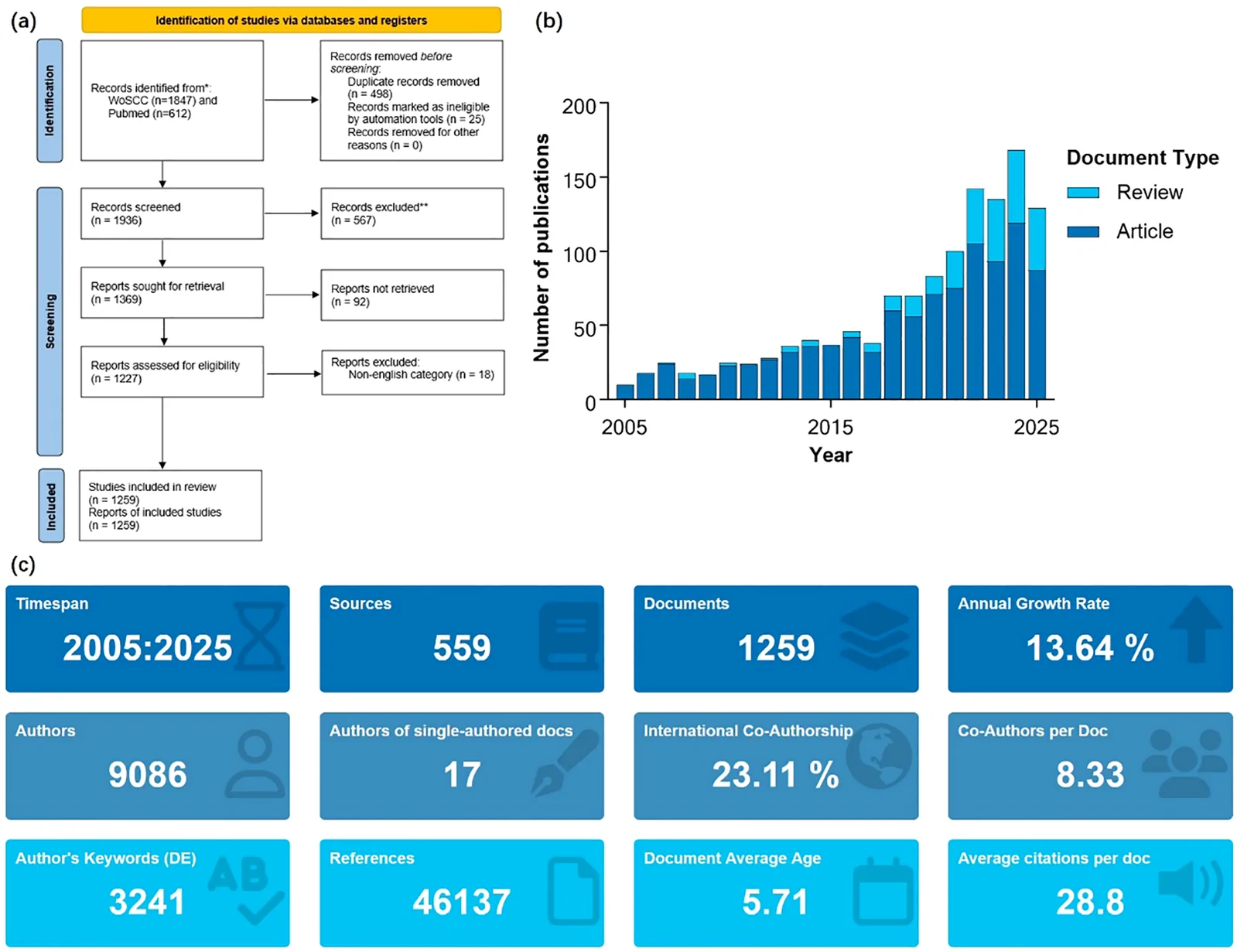
Bibliometric analysis of VR in cancer research: **(a)** PRISMA flow, **(b)** annual trends, **(c)** core metrics.

### 2.2 Bibliometric analysis and visualisation

Bibliometrics refers to an independent domain that permits quantitative reviews and analyses of available literature in a particular field (24). The results of the assessment include detailed information, such as authors, keywords, journals, countries, institutions, and references. Subsequently, visualisation is performed to establish internal correlations between the data procured, including similar topics that different authors have discussed, the research priorities of various institutions, and novel theories suggested by existing institutions. This study employed VOSviewer and CiteSpace for data analysis and visualisation. Country and regional co-occurrences, journal dual graph, high-frequency keyword trends, co-citations, literature citations surge, inter-country networks, and institutional researchers were analysed utilising the tools (23, 25). Thresholds were selected to balance network comprehensiveness and interpretability. Minimum publication thresholds of 10 for countries, 5 for institutions, and 30 co-citations for authors were applied, whilst keywords were retained with a minimum occurrence of 1. Clustering was performed using VOSviewer’s association strength algorithm and validated by silhouette scores, with CiteSpace modularity Q and mean silhouette scores confirming co-citation cluster validity. Duplicates were removed automatically by DOI and title matching in CiteSpace, followed by manual verification.

## 3 Results

### 3.1 Quantitative analysis of publication

This study identified 1,259 publications related to VR from 2005 to 2025. Among the publications, 1,002 were articles, whilst 257 were review articles (**Figure 1a**). According to the findings, the year-on-year publications in the field evaluated demonstrated a fluctuating trend, where a relatively high level was recorded in 2024 with 184 publications. Conversely, the number of publications up to September 2025 was lower than that of 2024 (**Figures 1b, c**).

### 3.2 Country and institutional analysis

The 1,259 articles identified in this study were published by 110 countries (**Figure 2a**; **Table 1**) and 2677 institutions (**Figure 2b**; **Table 2**). North American, European, Asian, and Oceanian nations were amongst the top 10 contributing countries, recording two, five, two, and one publications, respectively. Meanwhile, the United States of America (USA) and China had the largest number of publications, with 354 and 239, respectively. The most cited publications were also from the USA, with 9843 total citations and an average of 27.8 citations per article. Conversely, Pakistan had the lowest average citation frequency of 2.8 citations per paper and 14 citations for 5 publications. The highest average citation frequency was recorded by Jordan, where five publications received 226 citations, averaging 45.2 citations per paper.

**Figure 2:**
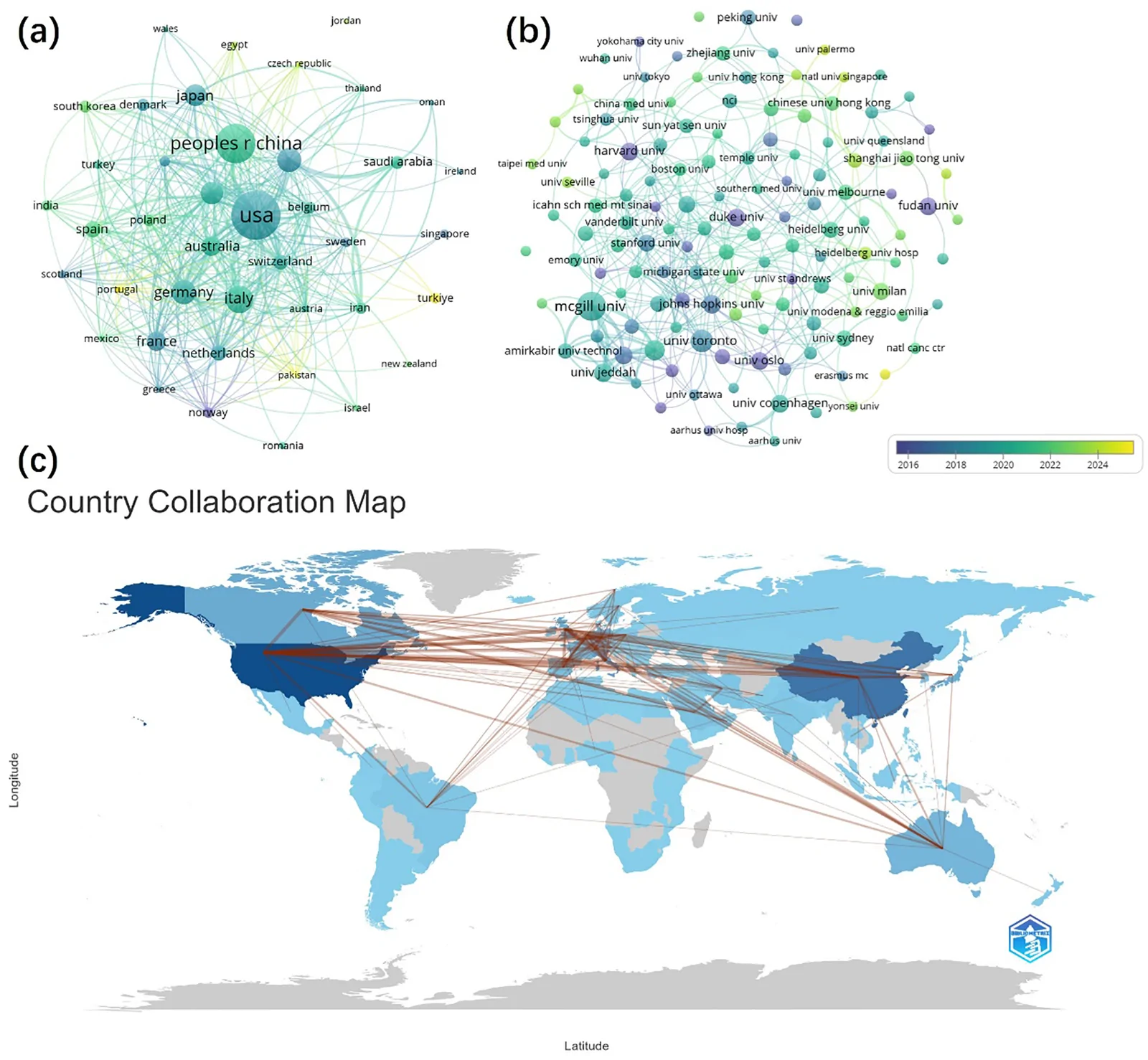
The distribution of publications from different **(a)** countries or regions and **(b)** organisations, and the **(c)** country collaboration map. The thickness of the connecting lines between the nodes reflects the strength of the interaction, while the colour of the node denotes the average year of publication for the country that the node represents.

**Table 1:** The top 10 most productive countries.

Rank	Country	Count	Proportion	Citation frequency	ACI	Total link strength
1	Usa	354	28.12	9843	27.81	236
2	China	239	18.98	3488	14.59	93
3	Italy	118	9.37	3014	25.54	124
4	Germany	95	7.55	2410	25.37	133
5	Canada	94	7.47	3624	38.55	112
6	England	83	6.59	2149	25.89	155
7	Japan	79	6.27	1422	18.00	40
8	France	62	4.92	1701	27.44	55
9	Australia	60	4.77	1287	21.45	82
10	Spain	44	3.49	689	15.66	54

ACI, Average Citation per Item.

**Table 2:** The top 10 most productive institutions.

Rank	Institution	Count	Proportion	Citation frequency	ACI	Total link strength
1	Mcgill Univ	35	2.78	1132	32.34	82
2	Univ Toronto	22	1.75	1052	47.82	22
3	King Abdulaziz Univ	17	1.35	455	26.76	59
4	Johns Hopkins Univ	15	1.19	386	25.73	19
5	Univ Jeddah	15	1.19	404	26.93	59
6	Fudan Univ	14	1.11	276	19.71	3
7	Harvard Univ	14	1.11	605	43.21	9
8	Duke Univ	13	1.03	604	46.46	9
9	King Fahad Med City	13	1.03	426	32.77	50
10	Univ Copenhagen	13	1.03	360	27.69	15

ACI, Average Citation per Item.

This study conducted visualisation for 38 countries. During the process, a minimum of 10 publications per country (**Figure 2c**) was set as the filter criterion. Resultantly, a considerable collaboration was observed between the nations. The outcomes also revealed that the top 10 institutions were primarily based in the USA (*n* = 3), Saudi Arabia (*n* = 3), Canada (*n* = 2), China (*n* = 1), and Denmark (*n* = 1). The information highlighted the increasing globalisation and diversity of VR interventions in tumour research. Furthermore, certain countries have gained a significant position in the competitive field by utilising their professional knowledge or participating in international cooperation. Meanwhile, visualisation according to the institution revealed McGill University with the highest number of publications (*n* = 35), followed by the University of Toronto (*n* = 22). The procedure was performed for 132 institutions with a minimum of five publications. The leading institutions also demonstrated substantial collaboration.

The uneven geographical distribution indicates gaps in research resources and funding across regions. High-output developed countries benefit from early investment in medical virtual reality platforms, whilst many emerging research countries expand their research capacity mainly through international collaboration. Close cross-institutional and cross-country cooperation will further accelerate the clinical translation of VR interventions for cancer in future studies.

### 3.3 Authors and co-cited authors

Based on **Figure 3a**, 168 authors were involved in the research collaboration network. According to publication count, Winkler-Schwartz and Del Maestro were amongst the top 10 authors (**Table 3**). Winkler-Schwartz authored 25 publications and recorded 931 citations, averaging 37.24 citation frequency. Meanwhile, Del Maestro averaged 47.38 in citation frequency, where his 21 publications received 995 citations. Nonetheless, Andrea documented the highest average citation frequency of 161 citations per publication. The author published four publications, which were cited 644 times.

**Figure 3:**
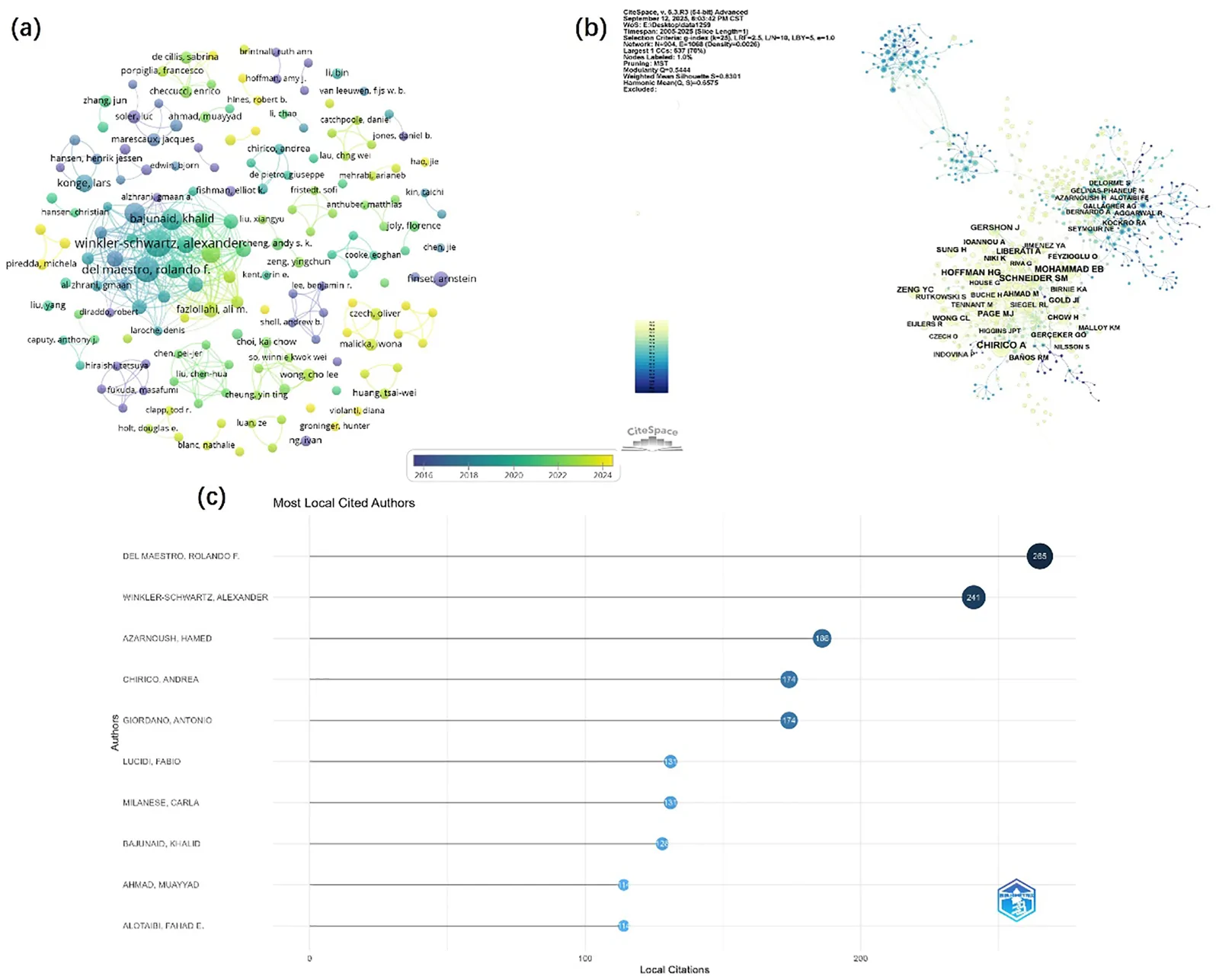
The author collaboration and citation landscape: the **(a)** author collaboration network, **(b)** author-related citation network, and **(c)** most locally cited authors. The thickness of connecting lines between nodes reflects the strength of interaction, and the node colour indicates the average publication year for the corresponding entity.

**Table 3:** The top 9 most productive authors.

Rank	Author	Count	Proportion	Citation frequency	ACI	Total link strength
1	Winkler-Schwartz Alexander	25	1.99	931	37.24	181
2	Del Maestro Rolando	21	1.67	995	47.38	109
3	Bajunaid Khalid	15	1.19	420	28.00	127
4	Azarnoush Hamed	14	1.11	538	38.43	88
5	Yilmaz Recai	12	0.95	462	38.50	88
6	Sabbagh Abdulrahman	12	0.95	350	29.17	100
7	Konge Lars	10	0.79	305	30.50	11
8	Ledwos Nicole	9	0.71	459	51.00	75
9	Alotaibi Fahad	9	0.71	272	30.22	70

ACI, Average Citation per Item.

**Figure 3b** illustrates the co-cited author network visualisation comprising 904 authors. This study only involved 25 co-cited authors during the visualisation, who had a minimum of 30 co-citations. Based on the results, Gershow occupied the most significant nodes, indicating close associations. For instance, Gershon collaborated closely with several other authors, including Ioannou, Jimenez, Liberati, and Feizioglu. Two authors in the top 10 co-cited authors have also been cited over 80 times: Chirico with 116, followed by Schneider with 85.

The most locally cited authors are represented in **Figure 3c**, where the horizontal axis denotes the amount of local citations and the vertical axis lists the authors. Among the authors identified in the findings, Del Maestro reached 265 local citations, which was the highest number, followed by Winkler-Schwartz with 241. The pattern revealed considerable differences in local citation frequencies across authors. Several prominent researchers also demonstrated remarkable influence in local scholarly discourse.

It is worth noting that high citation impact does not always correspond to large publication volume. A small number of core scholars shape the research agenda of this field, and their work provides critical references for follow-up exploratory studies.

### 3.4 Journals and co-cited journals

The 1259 publications analysed in this study were distributed across 560 journals. Nevertheless, the Surgical Endoscopy and Other Interventional Techniques journal recorded the most publications. The output of the journal steadily rose from four in 2009 to 30 in 2025. The journal also held the leading position for a significant period.

Based on the results, Cancers and Supportive Care in Cancer journals demonstrated the fastest growth. Although both journals documented low outputs (≤7) before 2020, their growth rates accelerated substantially after 2020, yielding outputs reaching 26 in 2025. Meanwhile, other journals, including World Neurosurgery and Journal of Clinical Medicine, had a steady growth, recording 20 and 18 outputs in 2025, respectively (**Figure 4a**).

**Figure 4:**
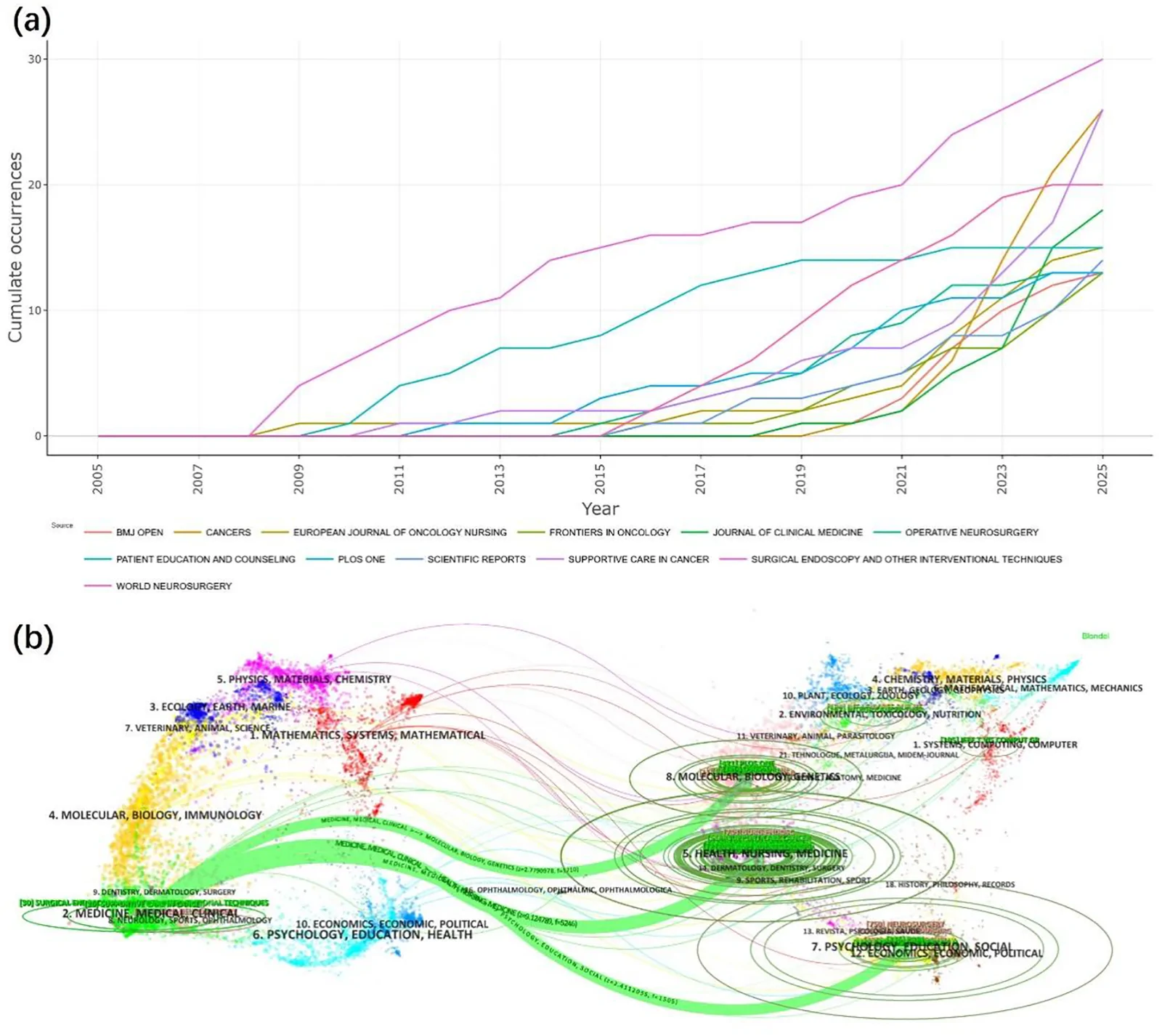
The trend publication quantity chart and **(a)** dual-map overlay of **(b)** relevant journals. Each node denotes a journal, the links are citation connectors representing an understanding of the interdisciplinary relationships in the field, and the colours reflect the different citation paths.

This study provided a journal dual-map overlay to illustrate the citation links between citing and co-cited journals. The citing journals cluster is demonstrated on the left side of the overlay, while the right side indicates the cited journals cluster. Based on the figure, the research covered multiple domains. For instance, publications in the medicine, medical, and clinical fields, which are denoted by the green path, predominantly cited literature from the molecular biology, genetics, health, nursing, medicine, psychology, education, and social domains. Similarly, other paths indicated that the publications in the veterinary, animal, and science fields frequently cite literature from the mathematics, systems, and mathematical domains, whilst research in ecology, earth, and marine typically cite literature from the physics, materials, and chemistry disciplines (**Figure 4b**).

### 3.5 Co-cited references

Citation burst references refer to instances of notable citation increments within a particular time frame. The data indicate a rise in interest from scholars in a certain discipline (**Figure 5a**). This study identified 25 references with significant citation bursts. Each bar in **Figure 5a** represents a year, and the red segments indicate considerable citation burst intervals (26).

**Figure 5:**
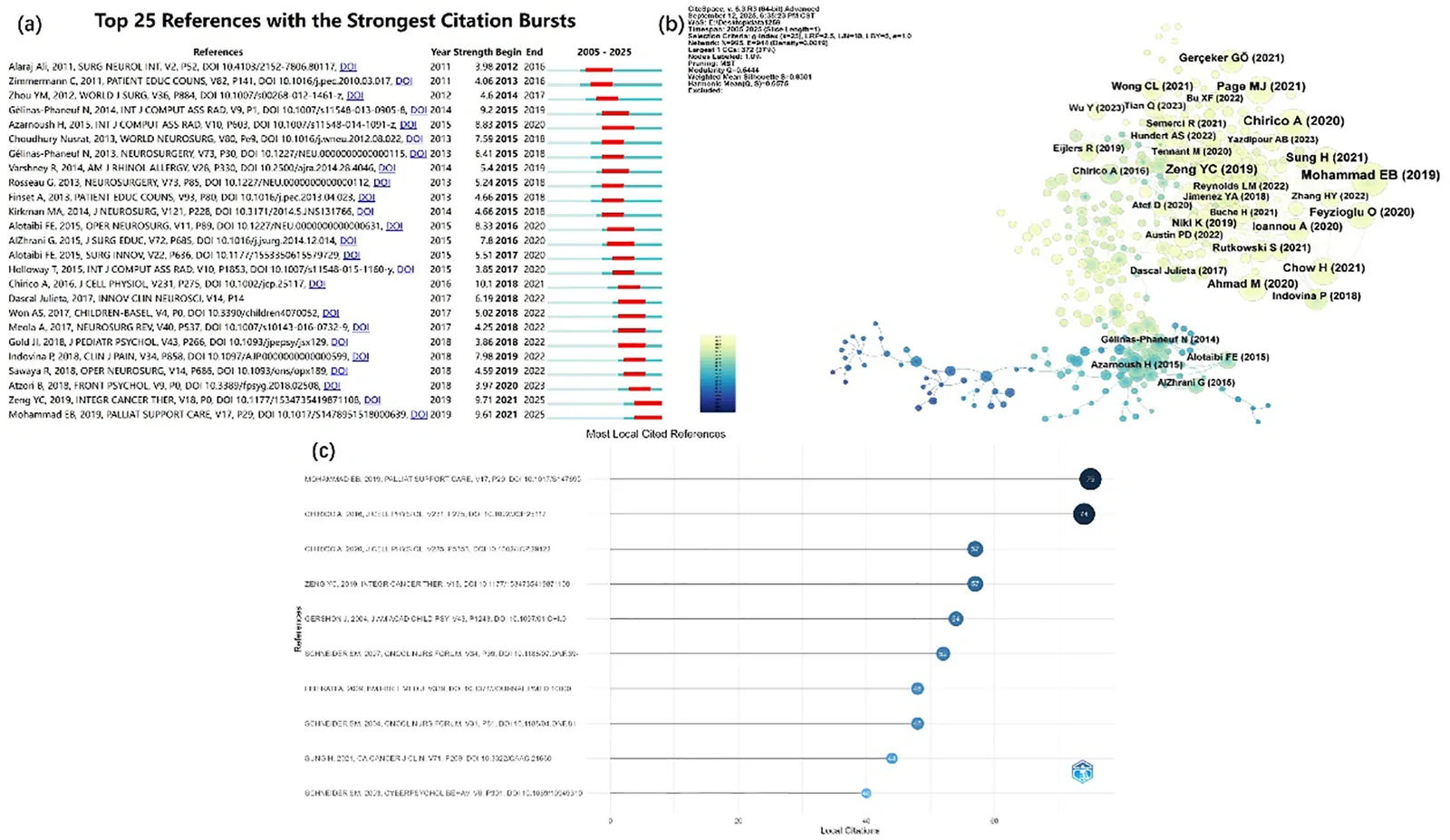
The bibliometric data on the top 25 references with **(a)** the strongest citation bursts, **(b)** the collaborative network, and **(c)** the most locally cited. The thickness of connecting lines between nodes represents the strength of interaction, while node colour demonstrates the average publication year for the corresponding entity.

Based on the findings, the earliest citation burst was recorded in 2012, whilst the most recent occurred in 2025. Chirico (11) documented the reference with the strongest citation burst in J CELL PHYSIOL in 2016. The citation intensity was maintained from 2018 to 2021. Meanwhile, the report by Zeng (27) published in INTEGR CANCER THER had a 9.71 burst intensity from 2021 to 2025. Overall, the 25 references demonstrated burst intensities ranging from 3.85 to 10.1 for two to three years.

In the field of cancer rehabilitation, 995 references were co-cited between 2005 to 2025. Based on **Figure 5b**, the top 10 co-cited references were cited at least 28 times. Among these references, the study by Mohammad (15) was the most cited locally, totalling 75, followed by Chirico (11), with 74 (**Figure 5c**).

References with citation bursts mark landmark turning points of this research domain. The duration of citation bursts suggests that hot topics in medical applied research usually attract concentrated attention for 2-3 years before evolving into new research directions.

### 3.6 Hotspots and frontiers

The keyword “virtual reality” (*n* = 596) emerged as the primary objective of cancer treatment when the co-occurrence of author keywords was assessed in this study. **Figure 6a** illustrates the network diagram obtained by filtering author keywords with a frequency of a minimum 1 occurrence. The findings also revealed breast cancer (*n* = 91), paediatric cancer (*n* = 80), brain cancer (*n* = 68), and lung cancer (*n* = 44) as the most commonly studied cancers. Meanwhile, VR (*n* = 596) and augmented reality (AR) (*n* = 110) were the focus of more extensive studies regarding treatment stages and the predominant intervention methods highlighted. In the context of cancer treatment, symptoms, such as cancer pain (*n* = 122) and anxiety (*n* = 84), have been gaining notable interest.

**Figure 6:**
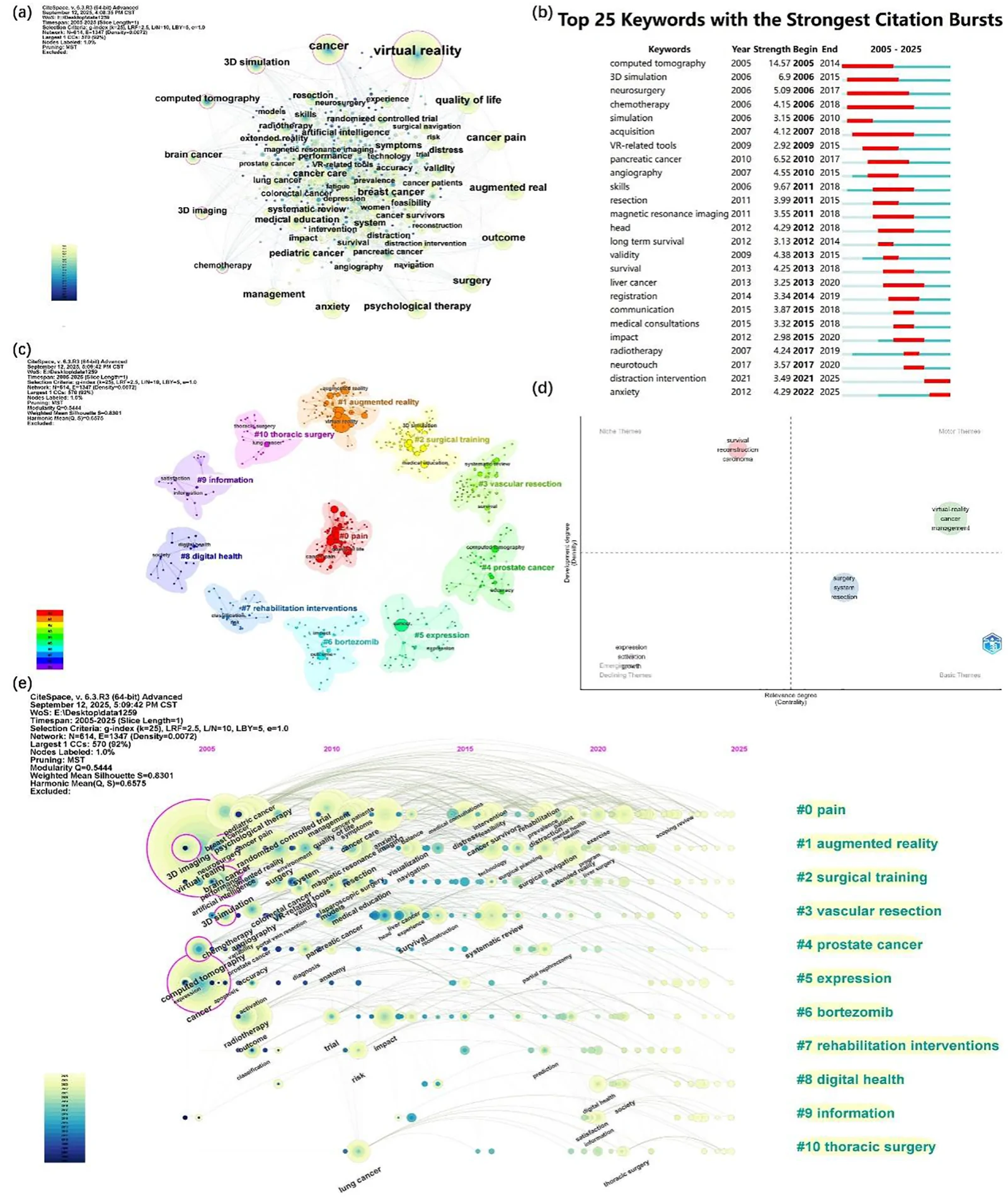
The keywords analysis results in oncology research demonstrating **(a)** keyword co-occurrence network, **(b)** top 25 keywords with the strongest citation bursts, **(c)** keyword clustering map, **(d)** additional keyword visualisation, and **(e)** thematic evolution. The connection and node attributes indicate keyword interaction and related characteristics, and for other subplots, visual elements correspond to applicable citation strength, clustering categories, or temporal dynamics.

This study analysed the top 25 keywords employed in the publications identified. The results demonstrated in **Figure 6b** revealed that “computed tomography” was the keyword with the highest intensity, achieving 14.57 from 2005 to 2014. Nevertheless, “anxiety” became the most intense keyword from 2022 to 2025.

The primary research clusters in the cancer domain are illustrated in **Figure 6c**. Ranked by importance, the most notable key cluster is pain, followed by AR and surgical training. In **Figure 6d**, the themes are categorised into motor-related (VR cancer management) and niche themes, whilst the evolution trend of the theme is demonstrated in **Figure 6e**. During the early stages, research was focused on the basic application of technologies, such as 3D imaging and computed tomography, in cancer diagnosis and treatment. Nonetheless, digital innovations, such as the integration of AR, surgical training, and digital health, gradually emerged as frontiers due to technological advancements. Precision medicine also continued to deepen to include subtype-specific therapies, such as vascular resection, prostate cancer management, and advancements in thoracic surgery. Furthermore, informatics promotes interdisciplinary studies, including molecular expression, novel drug development, exercise interventions, rehabilitation, and data integration. Conclusively, research has consistently shifted from traditional diagnostic techniques to a diverse paradigm that incorporates technological innovation, subtype precision, and interdisciplinary collaborations.

## 4 Discussion

This study offered systematic information on the VR development landscape in cancer research from 2005 to 2025 by employing bibliometric and visual analysis techniques. The results, including the exponential growth in literature quantity, the identification of core research topics, and current research hotspots and frontiers, aligned with data from previous related research. New directions for future exploration in the area were also underscored.

### 4.1 Interdisciplinary development trends

Cumulatively, the annual volume of literature on VR in cancer studies has been steadily rising. The observation is consistent with the increasing pattern of incorporating VR technology into fields such as oncology, psychology, and digital health. Moreover, research regarding AR and mixed reality (MR) in tumour surgery applications demonstrated an attention surge. The phenomenon was promoted by technological progress and the demand for precision and patient-centred medical care for cancer patients.

After 2018, a peak in literature growth was observed in this study, coinciding with the commercialisation and popularisation of consumer-grade VR devices. The increasing demand for psychological and functional rehabilitation of cancer patients also contributed to the outcome. The observations further underscore the role of technological accessibility in promoting translational research.

The results indicated that Winkler-Schwartz and Del Maestro from McGill University were amongst the authors who have received the highest interest in this field, producing H-Indexes of 21 and 52, and accumulated 52 and 284 publications, respectively. Both are also renowned scholars in their field. The authors played central roles in the field by focusing on research related to VR and oncology. They also predominantly explored VR technology for skill training and objective assessment in surgical oncology, particularly neurosurgical brain tumour (28, 29).

In a study, Winkler-Schwartz and Del Maestro developed a VR simulation platform for brain tumour resection. The authors confirmed that the simulator can realistically reflect the clinical operation scenarios by verifying its surface, content, and structural validity (30). Meanwhile, other reports by the authors (31–33) involved postoperative oncology rehabilitation, such as rehabilitation needs analysis for breast cancer and malignant limb bone tumours. The publications offered standardised and intelligent solutions for surgical oncology education and promoted the practical application of VR technology in clinical training.

The dominance of North America and Europe in VR cancer research can be attributed to three interrelated factors. First, these regions have substantially higher levels of public and private funding for digital health research, with agencies such as the National Institutes of Health in the United States and the European Research Council providing dedicated support for medical technology innovation. Second, advanced healthcare infrastructure, including widespread high-speed internet access, well-equipped academic medical centres, and established clinical trial networks, lowers the barrier for implementing and evaluating VR interventions. Third, greater digital health maturity in these regions means that clinicians and patients are more familiar with technology-enabled care, facilitating adoption and recruitment. In contrast, many low and middle income countries face constraints in hardware affordability, broadband connectivity, and trained personnel, which explains their lower research output despite growing interest in VR applications.

### 4.2 Research hotspots

Patients suffering from incurable cancers receive treatments that focus on prolonging their survival with minimal challenges to their quality of life (34). The VR has been suggested as an adjunctive treatment for reducing cancer-related symptoms (11), as cancer patients frequently stated that cancer treatment courses are painful and depressing. Keyword co-occurrence and topic prominence analyses also demonstrated “pain” as the core VR topic in cancer studies.

Seven randomised controlled trials (RCTs) were performed in the meta-analysis study by Chen et al. (35). Patients with various types of cancer, such as lung cancer, breast cancer, and colorectal cancer, were involved. According to the outcomes, the patients’ resting pain scores were notably reduced with VR intervention, recording a substantial effect size [SMD (95% CI) = −3.76 (−6.52, −1.00)]. Hoffman et al. (36) conducted a physical therapy randomised controlled study to establish the mechanism of VR in the treatment course for burn patients (**Figure 7**). Behavioural assessment and neurofunctional monitoring revealed that VR can capture individual attention through immersive 3D virtual environments, which included scenes with dynamic interactive tasks. The VR intervention activated the prefrontal cortex cognitive control network, actively shifting attention away from pain stimuli. Subsequently, abnormal neural activation in the insula (pain emotion integration centre) and anterior cingulate gyrus (pain signal transmission modulation area) was considerably inhibited. Endogenous opioid peptides were simultaneously triggered, enhancing analgesic effects. Therefore, a multidimensional synergistic effect of cognitive redirection-neural inhibition-endogenous substance regulation was observed. Furthermore, neuroimaging data confirmed that high-immersion VR (≥90°field of view angle) integrated with visual and auditory multisensory information can interfere with the convergence of thalamic pain signals. Resultantly, the functional connectivity of pain-related brain regions was diminished by 40%. The analgesic effect was also substantially better than traditional static visual distraction methods (37, 38).

**Figure 7:**
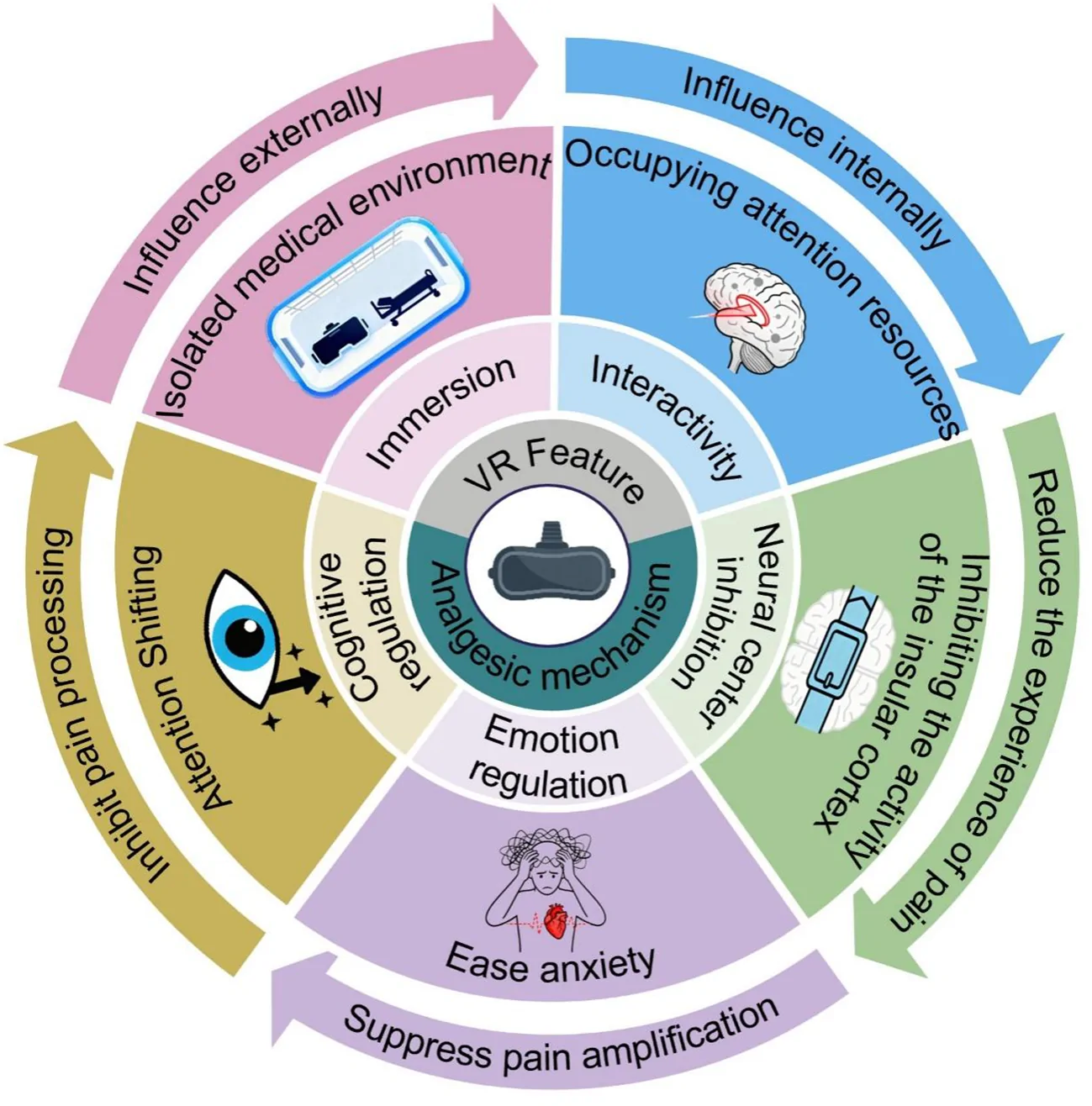
The overview chart of VR interventions to inhibit pain.

An underlying VR therapy attentional mechanism may derive the therapeutic effects of VR therapy in decreasing pain, anxiety, and depression levels (39). The VR systems limit human conscious attention, and the computer-generated multisensory input and interactive nature that the technology offers could make the patients feel like being transported into the VR world, leading to positive thoughts and emotions away from depression, pain, or anxiety (40).

The VR can be an innovative delivery tool for cancer patients with primary brain and other solid tumors (41). The technology can also be applied as targeted interventions to overcome the psychological barriers and pain suffered by breast cancer patients (42, 43). Nevertheless, available reports are significantly limited. Generally, the sample size documented by existing literature is small and employed predominantly single-centre designs. The research also focused on short-term analgesic effects, whilst the role of VR in long-term management of cancer pain remains unclear. Inadequate systematic assessments of the differences in the effects of VR scene types on analgesic effects are another limitation of available information. Conclusively, large-sample, multi-centre longitudinal studies are necessary for optimal VR scene and adaptation mechanisms for cancer patients.”Surgical training” and “vascular resection” were the topic clusters observed in this study. The data indicated the core role of VR in complex tumour surgery simulation. The randomised controlled trial by Valdis et al. (44) also confirmed that VR training can significantly improve operational efficiency despite the study focusing on robotic heart surgery. In the report, the VR group completed standardised surgical tasks at 20% to 30% less time than its non-VR training counterpart. The intraoperative operation score of the VR group also increased by over 100%.

The mechanisms of VR surgery simulation provide a reference for training in delicate operations, including vascular resections, which apply to complex surgeries, such as hepatobiliary tumours. In the field of pancreatic tumour surgery, VR can accurately replicate the vascular anatomical structure and resection scenario, and meet the clinical requirements of vascular resection (45). The technology can also facilitate physicians in improving their understanding of vascular proximity and reducing the risk of intraoperative injury. Furthermore, VR can simulate virtual operating room scenarios, which optimise surgery collaboration processes, anaesthesia, and nursing teams, and reduce the required period for unexpected situations during surgery.

The rise of “digital health” has driven the significant incorporation of VR into telemedicine, artificial intelligence (AI), and wearable devices, hence gradually building a digital ecosystem for cancer management courses. Relevant research have also validated the effectiveness of combining VR with multi-technology intervention in cancer patient management. For instance, Horesh et al. (46) conducted a pilot study on 42 breast and ovarian cancer patients. After 24 days of an AI-driven VR system intervention, the patients’ daily hot flash frequency significantly decreased. Improved stress, psychological distress, and sleep difficulties were also documented, with enhanced scores in quality of life-related dimensions. Furthermore, the patients were exceptionally satisfied with the system. The findings confirmed the feasibility of VR-AI in managing cancer-related symptoms.

While keyword analysis identifies AI and AR as emerging frontiers, their actual integration into cancer care remains at an early stage. For AR, clinical implementation has progressed furthest in surgical navigation and interventional oncology, where AR-guided systems have demonstrated noninferior accuracy compared with CT-guided localization for lung cancer resection and improved targeting precision for liver tumour ablation (47, 48). In radiation oncology, AR-based patient education and positioning systems have shown feasibility in reducing pretreatment anxiety and setup errors (49). For AI integration, the most advanced applications involve AI-driven VR systems that adapt intervention content in real time based on patient responses, such as the VR-AI platform that reduced hot flash frequency and improved psychological well-being in breast and ovarian cancer patients (46). AI is also being integrated into VR-based cognitive assessment tools for cancer patients, enabling automated scoring of attention, memory, and executive function. However, most AI-VR integration studies remain small scale pilots, and large scale validation, regulatory clearance, and cost-effectiveness evidence are still needed before routine clinical adoption.

Despite these advances, several implementation barriers remain. A 2026 meta-analysis confirmed significant effects of immersive VR on anxiety, depression, and fatigue in chemotherapy patients, but noted low evidence certainty due to protocol heterogeneity (50). Patient-level barriers include hardware cost, limited accessibility, and cyber sickness, whilst a 2025 mixed-methods study found that 64% of oncology clinicians identified cost as a key obstacle, alongside concerns about workflow integration and staff training (51). System-level challenges include insufficient infrastructure in resource-limited settings, lack of standardised protocols, and absent reimbursement policies. Addressing these multilevel barriers is critical for translating efficacy evidence into routine practice.

### 4.3 Research limitations

This study was the first to analyse cancer-related VR intervention research during the previous two decades by applying bibliometric visualisation methods. Nonetheless, this study had certain limitations. Firstly, the data were obtained only from the WoSCC and PubMed databases, whilst Scopus, Embase, and the Cochrane Library were not included. Although this two-database approach is consistent with many published bibliometric studies and captures the core oncology literature, it may omit records indexed exclusively in other databases. Although the WoSCC and Pubmed database offers comprehensiveness and reliability, it might not have all published articles. Additionally, the search strategy was restricted to “virtual reality” and “VR” as core terms, which may have omitted studies that exclusively use synonymous terms such as “extended reality” or “immersive technology” without mentioning virtual reality. However, given that virtual reality is the standard and most widely used term in this field, the potential impact on the overall bibliometric landscape is expected to be minimal. Another restriction of this study is only analysing literature published in English, which could potentially result in bias in the results. The data procured might also exhibit inconsistencies in various aspects, as the same institution might have employed various names at different times, and similar authors might be associated with varying institutions at different periods.

### 4.4 Future research directions

Future research should first deepen the mechanistic understanding of VR interventions by applying neuroimaging and behavioural science approaches to further analyse the specific mechanisms of VR in regulating pain, anxiety, and cognition in cancer patients, thereby supplementing the conceptual model proposed in this study. In parallel, expanding clinical verification through multi-centre, randomised controlled trials that adhere to clinical research guidelines is needed to systematically verify the effectiveness of VR across different cancer types and medical scenarios.

Over the longer term, research should integrate emerging technologies and assess the synergistic effects of VR, artificial intelligence, and wearable sensors to develop adaptive and personalised intervention programmes. Establishing standardised VR treatment protocols, generating cost-effectiveness evidence, and addressing implementation barriers such as hardware accessibility, clinician training, and reimbursement policies will also be essential to facilitate the routine integration of VR into oncology care.

## 5 Conclusion

This study employed various bibliometric analysis software to evaluate the current state of research on VR interventions in the field of cancer from different perspectives. Information on countries, journals, authors, institutions, and scientific categories are included in this study. The intrinsic relationships between keywords in varying clustering groups and their influences on the field are also specified. Moreover, the results revealed augmented reality, surgical training, molecular expression, new drug development, exercise interventions, and combined rehabilitation as the primary research topics that necessitated future research. Conclusively, this bibliometric analysis may provide answers for researchers involved in this field.

## Data Availability

The original contributions presented in the study are included in the article/**Supplementary Material**. Further inquiries can be directed to the corresponding author.
